# Antioxidant Effects and Cytoprotective Potentials of Herbal Tea against H_2_O_2_-Induced Oxidative Damage by Activating Heme Oxygenase1 Pathway

**DOI:** 10.1155/2020/7187946

**Published:** 2020-07-05

**Authors:** Ke-Xin Zhang, Jian-Bin Tan, Cheng-Liang Xie, Rong-Bo Zheng, Xiao-Dan Huang, Meng-Meng Zhang, Min Zhao

**Affiliations:** ^1^Center for Disease Control and Prevention of Guangdong Province, Guangzhou 511430, China; ^2^Department of Medical Statistics and Epidemiology, Guangdong Provincial Key Laboratory of Food, Nutrition and Health, Guangdong Engineering Technology Research Center of Nutrition Translation, School of Public Health, Sun Yat-Sen University, Guangzhou 510000, China; ^3^School of Pharmaceutical Science (Shenzhen), Sun Yat-Sen University, Guangzhou 510000, China; ^4^Guangzhou Wanlaoji Pharmaceutical Co., Ltd., Guangzhou 510403, China

## Abstract

Herbal tea with antioxidant ingredients has gained increasing attention in the field of functional foods due to their amelioration potential in aging-related diseases. Wanglaoji herbal tea (WHT) is a kind of traditional beverage made from herbal materials. This study was performed to investigate its antioxidant activity and identify its protective effect on a H_2_O_2_-induced cell damage model. In this study, we identified six kinds of phenolic acids with antioxidant activity in WHT, among which rosmarinic acid had the highest content and the highest contribution ratio to the antioxidant activity of WHT. Moreover, compared with the H_2_O_2_-induced damage group, the WHT treatment group can significantly increase the viability of cells and decrease the ratio of senescence-associated *β*-galactosidase-positive cells, intracellular malondialdehyde levels, and the percentage of G1 phase. Furthermore, enrichment analysis of differentially expressed genes revealed that heme oxygenase1 (HMOX1) was a key gene for protective effect of WHT on oxidative stress-induced cell damage. Thus, WHT exerted protective effects not only by scavenging reactive oxygen species but also by inducing the expression of cytoprotective genes by activating the HMOX1 pathway, which showed that WHT had a potential of promoting health by reducing oxidative stress-induced cell damage.

## 1. Introduction

Free radicals are active and unstable in nature due to unpaired electrons. In a human body, free radicals such as superoxide anion radical (O^2-^) and hydroxyl radical (OH^−^) are continuously produced as a result of normal cellular metabolism. They can serve various physiological functions such as energy production, phagocytosis, cell growth, and intracellular signaling. Normally, free radical is maintained in the moderate concentration by their own defense mechanism including antioxidative enzymes such as superoxide dismutase, catalase, and glutathione peroxidase and small molecule glutathione [1]. However, the concentration and/or activity of endogenous antioxidants will reduce with aging and cause overproduction of free radicals and the occurrence of oxidative stress, which will further result in cellular injury by damaging DNA, lipids, and protein [2, 3]. Therefore, the increase in oxidative stress has been related to the onset and progression of human aging. Accumulated evidences have showed that oxidative stress is a fundamental cause of aging-related pathologies and can induce the occurrence of aging-related disease such as cancer, diabetes, and cardiovascular and neurodegenerative diseases [4, 5]. These diseases have placed an enormous burden on the national health and welfare systems [1, 6]. It has been reported that antioxidant agents can reduce oxidative stress to help prevent aging-related disease progression [7]. Therefore, to prevent the occurrence of aging-related disease, additional antioxidant supplements will be necessary for the protection of cells from oxidative damage by neutralizing excessive free radicals.

Herbs have played a major role in traditional medicines for over a thousand years in China, which have a variety of health-benefit effects including antioxidant, anti-inflammatory, neuroprotective, and anticarcinogenic activities [1]. Many studies have demonstrated that the antioxidant activity of herbs is attributed to the bioactive composition such as flavonoids, alkaloids, and polyphenols [8, 9]. They display antioxidant activity by acting as reducing agents, hydrogen donators, and singlet oxygen quenchers [8]. Moreover, the natural antioxidants derived from herbs can activate cellular defense mechanisms to attenuate cellular oxidative stress and protect against aging-related diseases [10]. It has been reported that resveratrol can significantly attenuate oxidative damage in a cell model of Alzheimer's disease by decreasing oxidative status [11]; anthocyanins can protect from aging-related neurodegenerative disease through both direct and indirect antioxidant activities within the brain [12]. Therefore, herbs with antioxidative ingredients have gained increasing attention as antioxidant supplements to combat aging-related diseases.

Wanglaoji herbal tea (WHT), a water decoction of seven raw herbal materials, is a famous herbal beverage in south China, which originated in the Qing Dynasty and so far has a history of about 200 years. Previous study has reported that WHT has biological effects of immunomodulation and hepatoprotection, which can be contributed to phenolic compounds such as rosmarinic acid and caffeic acid [13]. It has been reported that rosmarinic acid can protect human liver cell line L02 from oxidative damage through suppressing cell apoptosis with the activation of the MAPK and Nrf2 signaling pathway [14]. Caffeic acid, a major metabolite of chlorogenic acid, can reduce oxidative stress and microglial activation in the mouse hippocampus [15]. However, the protective effect of WHT on the oxidative stress-induced cell damage during aging has not been elucidated. Young human fetal lung diploid fibroblast cell (2BS cell) is usually used as a cell model for antiaging evaluation because it can be caused by premature senescence by H_2_O_2_ [6]. To understand the antioxidant mechanism of WHT and develop its neutraceutical application for promoting health, we will identify the antioxidant compounds in WHT and investigate its ameliorative effect on H_2_O_2_-induced 2BS damage and the possible mechanism underlying this protective effect in the present study.

## 2. Materials and Methods

### 2.1. Materials

The extract of WHT was provided by Wanlaoji Pharmaceutical Co., Ltd. (Guangzhou, China), which is made from seven traditional Chinese herbs, which are *Mesona chinensis*, *Plumeria rubra*, *Microctis folium*, *Chrysanthemum indicum*, *Lonicera japonica*, *Prunella vulgaris*, and *Glycyrrhiza uralensis* (1 g of the extract is equivalent to 12.89 g of raw herbal materials). Caffeic acid, rosmarinic acid, 1,1-diphenyl-2-picrylhydrazyl (DPPH), and 2,4,6-Tri(2-pyridyl)-s-triazine (TPTZ) were purchased from Sigma-Aldrich Co. (St. Louis, MO, USA). Protocatechuic acid, isochlorogenic acid C, cryptochlorogenic acid, and neochlorogenic acid were purchased from the National Institute for the Control of Pharmaceutical and Biological Products (Beijing, China). HPLC grade methanol was obtained from Burdick & Jackson (Muskegon, MI, USA). All other reagents and chemicals were analytical grade.

### 2.2. UPLC-MS/MS Analysis

Quantification of all analytes was carried out using an ultrahigh performance liquid chromatography (UPLC) system (Agilent 1290) with an electrospray ionization (ESI) system coupled to a triple-quadrupole mass spectrometer (Agilent 6410, CA, USA). UPLC separation was performed with a C18 column (2.1∗150 mm, 1.9 *μ*m, Agilent Infinitylab Poroshell 120), and the mobile phase consisted of 0.1% formic acid in water (solution A) and 0.1% formic acid in methanol (solution B). The gradient of elution was as follows: 0-3.5 min, 3%-5% B; 3.5-7.5 min, 5%-18% B; 7.5-14 min, 18%-25% B; 14-14.5 min, 25%-45% B; 14.5-16 min, 45%-50% B; 16-21 min, 50%-95% B; 21-22 min, 95%-3% B; and 22-24 min, 3%-3% B. The flow rate and injection volume were 0.4 mL/min and 1 *μ*L, respectively. For mass data acquisition, the precursor ion and the product ions were selected based on the previous injection of standards. The choice of ions, collision energy, and fragmentation was optimized for each analyte. The electrospray system was operated in the negative mode at 4000 V, with the nebulizer pressure set to 50 psi, and the source temperature to 350°C. Nitrogen was used as nebulizer and collision gas. Data acquisition was performed in the multiple-reaction monitoring (MRM) mode. The precursor ions [M-H]^−^ and their most abundant product ions were selected to perform the analysis. All MRM parameters were summarized in Table S1.

### 2.3. Scavenging Ability on DPPH Radical

DPPH radical scavenging was measured as described by Xie et al. [16]. An aliquot (175 *μ*L) of 0.2 mM DPPH was mixed with 25 *μ*L test sample in 96-well microplates. After incubating at room temperature in the dark for 30 min, the absorbance was measured at 517 nm. The scavenging ability on DPPH radical was calculated as follows:

(1) $$\begin{array}{c} \text{Scavenging ability on DPPH radical}=\frac{A_{\text{conrol}}-A_{\text{sample}}}{A_{\text{control}}}*100, \end{array}$$

where *A*_control_ and *A*_sample_ are the absorbance of the control and the sample, respectively.

The concentration of decreasing the initial DPPH concentration by 50% (IC50) was calculated by the dose-response curve.

### 2.4. FRAP (Ferric Reducing Antioxidant Power) Assay

The FRAP assay was carried out as described by Wetchakul et al. [17]. A portion of an aqueous 10 mM solution of 2,4,6-Tri(2-pyridyl)-s-triazine (TPTZ) was mixed with the same volume of 20 mM FeCl_3_ and 10 times volume of acetate buffer (0.3 M, pH 3.6). Then, 180 *μ*L of the mixture was incubated with 20 *μ*L of test sample for 30 min at room temperature, and the absorbance was measured at 593 nm. Half effective concentration (EC50), at which the absorbance is equal to 0.5, was calculated by the standard curve of absorbance against extract concentration.

### 2.5. Cell Culture

Human embryonic lung epithelial diploid fibroblasts (2BS cells, 25-generation-old) were obtained from BeiNaChuangLian Biotechnology Institute (Beijing, China) and cultured in Dulbecco's modified Eagle medium (DMEM) supplemented with 10% fetal bovine serum (FBS) and 1% penicillin-streptomycin (Gibco, NY, USA) at 37°C in a humidified atmosphere containing 5% CO_2_.

### 2.6. Cell Viability

After subculture, 2BS cells (2∗10^5^ cells/mL) were seeded in 96-well plates and incubated for 24 h. After treatment with 800 *μ*M H_2_O_2_ alone and combined with different concentrations of WHT for another 24 h, cell viability was estimated by the MTT assay. Cell viability was expressed as the percentage of absorbance of the sample groups compared with that of the control group, which was in the absence of H_2_O_2_ and WHT.

### 2.7. Senescence-Associated *β*-Galactosidase (SA-*β*-gal) Activity

After 2BS cells (2∗10^5^ cells/mL) were seeded in 6-well plates and incubated for 24 h, cells were treated with 800 *μ*M H_2_O_2_ alone and combined with different concentrations of WHT for another 24 h. The SA-*β*-gal activity of cells was evaluated by the SA-*β*-gal staining kit (Solarbio, Beijing, China) according to the manufacturer's protocol. Briefly, after 1 mL staining fixative was added and incubated for 15 min at room temperature, the culture medium was discarded. Then, 1 mL *β*-galactosidase staining was added and incubated at 37°C for 24 h. The proportion of senescent cells was assessed by counting the percentage of SA-*β*-gal-positive cells using light microscopy.

### 2.8. Detection of Malondialdehyde (MDA)

The content of cellular MDA was measured by a Lipid Peroxidation MDA Assay Kit (Beyotime, Shanghai, China) according to the manufacturer's protocol. Briefly, the sample reacted with thiobarbituric acid (TBA) upon boiling and generated the resultant pink adducts, which were measured by a Varioskan Flash (Thermo Fisher Scientific Company Inc., Rockford, USA) at 532 nm. The content was calculated by a standard curve of MDA. Meanwhile, the protein content was determined by the BCA protein assay kit (Pierce Company). The content of MDA was expressed as MDA nmol/mg protein.

### 2.9. Cell Cycle Analysis

Flow cytometry was utilized to detect the change of cell cycle distribution according to the method of Li et al. [18]. Cells were harvested by trypsinization, washed with cold phosphate-buffered saline (PBS), and then fixed with ice-cold 70% ethanol at 4°C overnight. The cells were stained by the propidium staining assay kit (Beyotime, Shanghai, China) according to the manufacturer's protocol. The distribution of cell cycle was measured by a flow cytometer (FACSCalibur, Beckton Dickinson, USA), and the percentage of cells in G1, S, and G2 phases was analyzed by the software of FlowJo (Tree Star, Ashland, OR, USA).

### 2.10. RNA-seq Analysis

The RNA-seq assay was carried out as described by Ma et al. [19]. Total RNA was extracted using the TRIzol reagent (Invitrogen), and all the samples were sent to BGI Corporation (Shenzhen, China) for further RNA-seq analysis via the BGISEQ-500 platform (BGI, Wuhan, China). The raw data have been submitted to NCBI (BioProject ID: PRJNA601632; https://www.ncbi.nlm.nih.gov/bioproject/601632). Gene expression levels were calculated and expressed as Fragments per Kilobase per Million reads (FPKM) mapped. Genes with FPKM ≥ 1 were considered expressed and analyzed. Fold changes (FC) were considered as significant when the absolute value (Log_2_FC) is greater than 0.2, with a *p* value < 0.05. The functional enrichment analysis of differentially expressed genes (DEGs) was performed by Ingenuity Pathway Analysis (IPA) software (http://www.Ingenuity.com).

### 2.11. Statistical Analysis

Data were expressed as the mean and standard deviation of at least triplicate for each sample. One-way analyses of variance (ANOVA) were used to analyze the between-group differences. After this, GraphPad Prism 5 software (GraphPad Software Inc., La Jolla, CA) was used to carry out Tukey's HSD test.

## 3. Results

### 3.1. Content of Phenolic Acids in WHT and Their Antioxidant Activities

UPLC/MS were used to identify and assay the content of phenolic acids in WHT. The mass spectrum for tentative phenolic acids was shown in Fig. S1, which were consistent with retention time and the ion pairs of the corresponding standard materials. The identified phenolic acids included rosmarinic acid, caffeic acid, protocatechuic acid, isochlorogenic acid C, cryptochlorogenic acid, and neochlorogenic acid, among which the content of rosmarinic acid was the highest (3426.9 *μ*g/g), followed by isochlorogenic acid C, neochlorogenic acid, cryptochlorogenic acid, protocatechuic acid, and caffeic acid (Table 1).

DPPH and FRAP assays were applied to evaluate the antioxidant properties of phenolic acids. A dose-dependent relationship was found in DPPH scavenging activity within the range of test concentration (Fig. S2A). IC50 of different phenolic acids for DPPH radical scavenging activities were from 11.7 to 33.1 *μ*M, among which isochlorogenic acid C showed the highest activity, followed by rosmarinic acid, protocatechuic acid, caffeic acid, cryptochlorogenic acid, and neochlorogenic acid (Table 1); similar to the results in the DPPH assay, all phenolic acids were found to exhibit FRAP activity. Their activities were in a dose-dependent manner (Fig. S2B), and the EC50 varied from 10.0 to 26.8 *μ*M as represented in Table 1. Rosmarinic acid exhibited the highest activity on the FRAP assay, and the FRAP activity of six phenolic acids was found to be in the order of rosmarinic acid, caffeic acid, isochlorogenic acid C, cryptochlorogenic acid, protocatechuic acid, and neochlorogenic acid.

**Figure 1:**
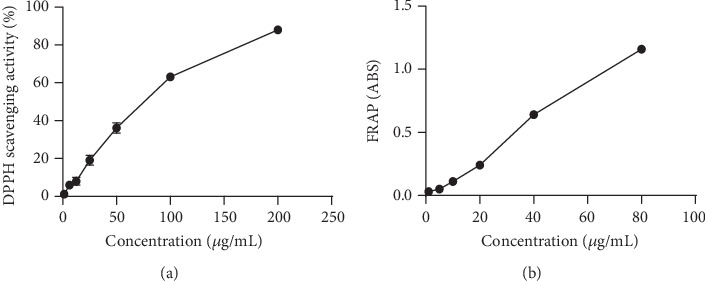
The antioxidant activity of Wanglaoji herbal tea determined by DPPH (a) and FRAP (b) assays at different concentrations.

The dose-response curves of WHT using DPPH and FRAP assay were shown in Figure 1, which showed that WHT had dose-dependent DPPH radical scavenging activity and FRAP activity. Moreover, for WHT, the IC50 of DPPH scavenging activity and EC50 of FRAP activity were calculated according to the corresponding dose-response curves, respectively (Tables S2 and S3). Considering the content of phenolic acids in WHT and their relative antioxidant activity to WHT, the contribution ratio of the individual phenolic acid to total antioxidant activity of WHT was from 0.2% to 4.8% for DPPH scavenging activity and from 0.4% to 8.3% for FRAP activity, respectively (Tables S2 and S3). The total contribution ratio of six phenolic acids was 9.6% on DPPH scavenging activity and 13.6% on FRAP activity, respectively. Thus, the antioxidant activity of WHT was partially attributed to the phenolic compounds, among which rosmarinic acid was the predominant antioxidant ingredient.

### 3.2. Protective Effect of WHT on H_2_O_2_-Induced Cell Damage

To establish a H_2_O_2_-induced cell damage model, the effect of H_2_O_2_ on the viability of 2BS was investigated. As shown in Fig. S3, the viability of cells significantly reduced after treatment with 800 *μ*M H_2_O_2_ for 24 h. Therefore, the conditions were used in subsequent experiments.

**Figure 2:**
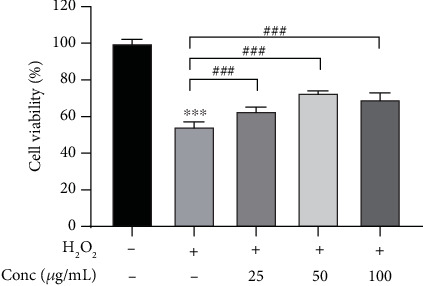
Wanglaoji herbal tea (WHT) reduced the loss of viability induced by H_2_O_2_ after treatment with H_2_O_2_ and/or different concentrations of WHT. ^∗∗∗^*p* < 0.001 versus without H_2_O_2_ treatment; ^###^*p* < 0.001 versus H_2_O_2_ treatment.

**Figure 3:**
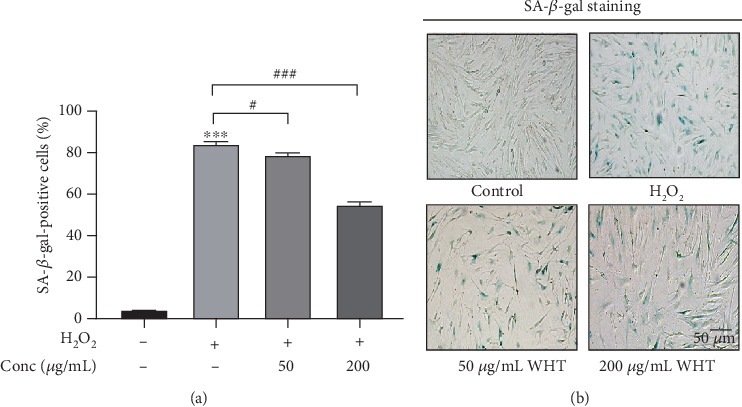
Wanglaoji herbal tea (WHT) attenuated H_2_O_2_-induced cellular senescence after treatment with H_2_O_2_ and/or different concentrations of WHT. (a) Percentage of senescence-associated *β*-galactosidase- (SA-*β*-gal-) positive cells; (b) images of SA-*β*-gal activity. ^∗∗∗^*p* < 0.001 versus without H_2_O_2_ treatment; ^#^*p* < 0.05, ^###^*p* < 0.001 versus H_2_O_2_ treatment.

**Figure 4:**
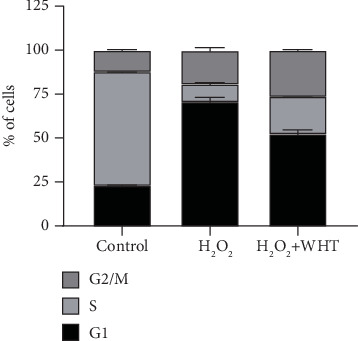
Cell cycle distribution of 2BS after treatment with H_2_O_2_ and/or Wanglaoji herbal tea (WHT) at the concentration of 200 *μ*g/mL.

**Figure 5:**
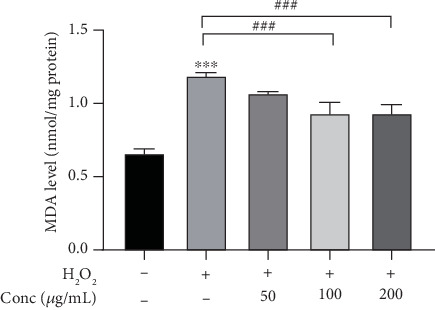
Wanglaoji herbal tea (WHT) decreased the malondialdehyde (MDA) level after treatment with H_2_O_2_ and/or different concentrations of WHT. ^∗∗∗^*p* < 0.001 versus without H_2_O_2_ treatment; ^###^*p* < 0.001 versus H_2_O_2_ treatment.

In order to avoid cytotoxic effect of WHT on subsequent experiments, first, the effect of WHT on cell viability of 2BS was investigated. No cell toxicity was observed when treated by WHT at test concentrations for 24 h (Fig. S4). When compared with the H_2_O_2_ group, the WHT-treated group significantly improved the cell viability from 54.5% to 73.0%, which suggested that WHT could partially reverse the H_2_O_2_-induced cell death (Figure 2). SA-*β*-gal staining was used to label premature senescent cells. In the control cells, only 4.2% cells were positive. After treatment with H_2_O_2_, a significant increase of SA-*β*-gal-positive cells (84.1%) was observed compared with the control group. After treatment with WHT, the percentage of SA-*β*-gal-positive cells showed a declining dose-dependent tendency, in which the ratio decreased to 54.9% at the concentration of 200 *μ*g/mL (Figure 3). Furthermore, the cell cycle phase distribution was examined. Cells treated with H_2_O_2_ exhibited a significantly increased proportion in the G1 phase as the proportion of cells in the G1 phase was 70.9% compared to 23.3% in the control. WHT treatment eliminated the effect of H_2_O_2_ and reduced the proportion of cells in the G1 phase to 52.7%, which showed that WHT protect against H_2_O_2_-induced senescence (Figure 4). To further assay the antioxidative effect of WHT, the most frequently used oxidative stress-related biomarker MDA was detected. H_2_O_2_ treatment significantly increased the cellular MDA level from 0.66 to 1.19 nmol/mg protein, which was effectively decreased in a dose-dependent manner after treatment with WHT (Figure 5). Together, the current data suggested that WHT can attenuate the oxidative stress-induced cell damage in 2BS.

### 3.3. Transcriptome Profiling of Gene Expression and Functional Enrichment Analysis of DEGs

**Figure 6:**
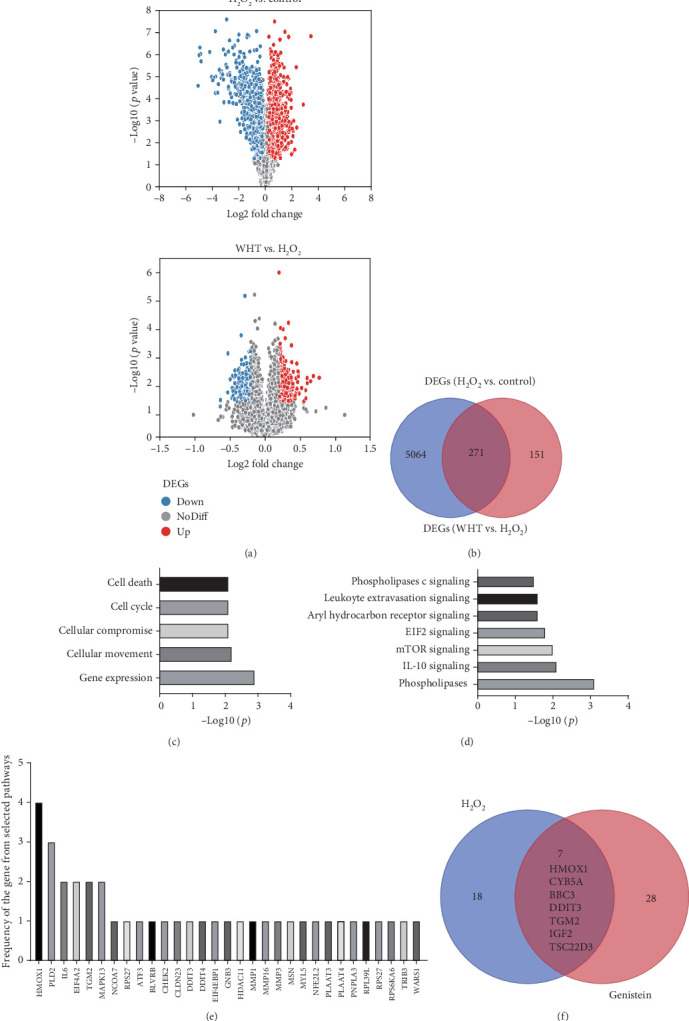
Transcriptomic alterations in 2BS cells upon different treatments by vehicle (control), H_2_O_2_, and Wanglaoji herbal tea (WHT). (a) Volcano plot of significant and nonsignificant differentially expressed genes upon different treatments (red or blue colors indicates the up- and downregulated genes, respectively); (b) Venn diagram comparison of H_2_O_2_ treatment vs. control and WHT treatment vs. H_2_O_2_ treatment; (c) significant molecular and cellular function identified by Ingenuity Pathway Analysis (IPA) software; (d) significant canonical pathway identified by IPA software. (e) Frequency analysis of differentially expressed genes from enriched signaling pathway; (f) Venn diagram comparison of the genes which were regulated by WHT in the same direction by regulators from the mechanism network of genistein and opposite direction by regulators from the mechanism network of H_2_O_2_ by an upstream regulator analysis tool of IPA.

RNA-sequencing generated approximately 21.8 million 50 bp raw reads, the average of the clean read rate was 97.9%, and the average of the mapping rate was 95.3% (Table S4). According to the selection criteria for DEGs, 5335 DEGs were identified between the control group and the H_2_O_2_ group; 422 DEGs were identified between the WHT group and the H_2_O_2_ group (Figure 6(a)). Moreover, 271 common DEGs were found, among which 163 genes were upregulated and 108 genes were downregulated significantly after treatment with WHT compared with the H_2_O_2_ group (Figure 6(b) and Table S5). In order to identify the genes and pathways in response to the antioxidant activity of WHT, functional enrichment analysis was performed by IPA software. Based on the result from IPA analysis, molecular and cellular functions of 271 common DEGs were associated with cell death and survival, cellular movement, cell cycle, gene expression, and cellular compromise (Figure 6(c) and Table S6). Based on IPA Knowledge Base, the enriched canonical pathways were phospholipases C signaling, leukocyte extravasation signaling, aryl hydrocarbon receptor signaling, EIF2 signaling, mTOR signaling, IL-10 signaling, and phospholipases (Figure 6(d)). Through analyzing the frequency of genes involved in these signaling pathways, HMOX1 was found as an important core gene (Figure 6(e) and Table S7). Additionally, genistein (an antioxidant compound) and H_2_O_2_ were found as significant upstream regulators for common DEGs by the Upstream Analysis of IPA, and the predicted mechanism networks regulated by genistein and H_2_O_2_ were showed in Figs. S5 and S6. According to the predicted mechanism networks and the regulation of regulators from mechanism networks to DEGs, those DEGs were selected as antioxidant activity-related genes which were regulated by WHT in the same direction by regulators from the mechanism network of genistein and opposite direction by regulators from the mechanism network of H_2_O_2_ (Tables S8 and S9). Seven genes HMOX1, CYB5A, BBC3, DDIT3, TGM2, IGF2, and TSC22D3 were found from DEGs, which may be related to antioxidative activity of WHT (Figure 6(f)). In combination with the results from canonical pathway and upstream regulator analysis, HMOX1 was proposed as a key gene for the cytoprotective effect of WHT against oxidative stress.

## 4. Discussion

Oxidative stress is a risk factor associated with aging-related diseases, which usually is caused by overproduction of reactive oxygen species (ROS) and/or damage to the antioxidant system [20]. Traditional herb has been applied clinically in China for over 2000 years. Recently, it has attracted much attention worldwide in the field of aging intervention relying on their large variety of naturally active chemicals and bioactive activities [21]. WHT is a famous herbal beverage in south China. It has been reported that WHT contained antioxidant ingredients such as phenolic compounds [13]. Therefore, we hypothesized that WHT can play a role in promoting health by preventing the overproduction of ROS.

Phenolic compounds are polyhydroxylated compounds, constituting one of the most extensive groups of naturally active chemicals and exhibiting a variety of biological functions such as protection against oxidative stress-induced diseases [22]. In this study, we identified six phenolic acids from WHT, and they all exhibited high antioxidant activity, which is consistent with the results from previous research [14, 23, 24]. Among these phenolic acids, rosmarinic acid had the highest content and the highest contribution ratio to the antioxidant activity of WHT, which is a major component of *Prunella vulgaris* (a raw material of WHT) [25]. However, total antioxidant activity of all six phenolic acids only contributed to 9.6% of DPPH radical scavenging activity and 13.6% of FRAP activity of WHT. Therefore, the antioxidant activity of WHT may be partially contributed to the identified phenolic acids, and the antioxidant compounds in WHT needed to be investigated in the future.

Oxidative stress is caused by an imbalance between reduced and oxidized biomolecules within cells [26]. It has been reported that H_2_O_2_ was efficient in inducing cell damage including premature senescence and cell death by the production of oxidative stress [2, 6]. Exposure to H_2_O_2_ has become a classic model to investigate the oxidative stress susceptibility and antioxidant activity of compounds [27]. Previous study has shown that exposure to H_2_O_2_ could induce intracellular ROS accumulation and inhibit DNA synthesis and cell proliferation resulting in loss of cell viability [28]. Moreover, H_2_O_2_-induced cell damage can result in the activation of cell cycle checkpoint proteins and initiation of the process of DNA repair. During the recovery time, the unrepaired damaged DNA in the cell led to premature senescence. SA-*β*-gal activity is a biomarker of premature senescence of 2BS cells [6]. We found that the ratio of SA-*β*-gal-positive cell and percentage of G1 phase was significantly increased after cells were subjected to H_2_O_2_, which was in accordance with the previous report [6, 28]. During oxidative stress, ROS can trigger peroxidation of membrane lipids resulting in the formation of MDA. Therefore, MDA is closely related to oxidative stress status of cells and has been widely used as a biomarker of oxidative stress [29]. The cellular MDA level was usually maintained at a low level in normal status but significantly increased after treatment by H_2_O_2_ [30]. In this study, we tested the effects of WHT on H_2_O_2_-induced 2BS cells. Compared with the H_2_O_2_-treated group, WHT can significantly increase the viability of cells and decrease intracellular MDA levels and the ratio of SA-*β*-gal-positive cells and the percentage of G1 phase in a dose-dependent manner, which showed that WHT can protect 2BS from H_2_O_2_-induced cell damage.

**Figure 7:**
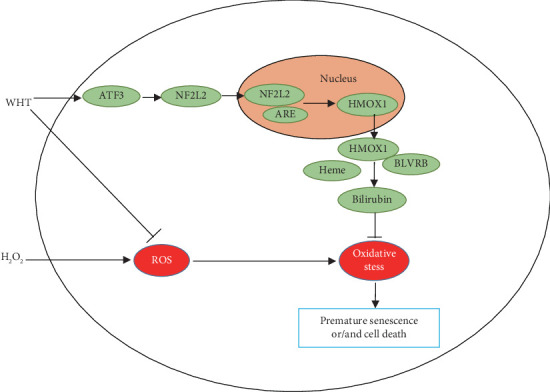
Schematic of proposed cytoprotective mechanism of Wanglaoji herbal tea (WHT) against H_2_O_2_-induced cell damage in 2BS cells.

RNA-seq analysis was used to investigate the molecular mechanisms involved in WHT-mediated antioxidant effects. Based on the result from the canonical pathway and upstream regulator analysis by IPA, HMOX1 was identified as a key gene for protection against oxidative stress. HMOX1 is an important rate-limiting enzyme in heme catabolism and widely distributed in mammalian tissues, which not only is an oxidative stress marker but also has some cytoprotective properties [31]. It has been shown that HMOX1 knockout mice produced oxidative tissue injury and chronic inflammation [32]. Moreover, HMOX1 converts heme to biliverdin, which can be further degraded to bilirubin by BLVRB to exert antioxidant activities [33–35]. Previous study has showed that the NFE2L2-ARE signaling pathway plays critical roles in maintaining the cellular redox balance [36]. However, in the NFE2L2-ARE signaling pathway, NFE2L2 does not elicit an antioxidant effect but rather helps upregulate the expression of antioxidant enzyme HMOX1 [37]. Additionally, it has been reported that NFE2L2/HMOX1 signaling can be activated by ATF3 [38]. In this study, WHT treatment significantly enhanced the expression of cytoprotective gene HMOX1 and HMOX1 related upstream and downstream genes such as ATF3, NFE2L2, and BLVRB. So, the cytoprotective effect of WHT may be attributed to the activation of the HMOX1 pathway (Figure 7), but it still needs to be investigated in greater detail in the future.

## 5. Conclusions

WHT had antioxidant activity, which can be partially contributed to the phenolic acids, and it showed cytoprotective effect on H_2_O_2_-induced 2BS cells by increasing the cell viability, reducing the MDA level, and decreasing the ratio of SA-*β*-gal-positive cells and the percentage of G1 phase. WHT exerted protective effects not only by scavenging ROS but also by inducing the expression of cytoprotective genes by activating the HMOX1 pathway. Our findings laid the groundwork for elucidating the antioxidant chemical constituents and understanding the underlying protective mechanism of WHT on oxidative stress-induced cell damage. To promote the utilization of WHT in maintaining health, we will further investigate whether the identified bioactive ingredients in WHT have synergic protect effect on H_2_O_2_-induced cell damage and perform *in vivo* experiments to evaluate the ameliorative role of WHT in aging-related disease in the future.

## Figures and Tables

**Table 1:** Content and antioxidant activity of phenolic acids in Wanglaoji herbal tea.

Phenolic acid	Content (*μ*g/g)	Antioxidant activity (*μ*M)	
		DPPH (IC50)^1^	FRAP (EC50)^2^
Rosmarinic acid	3426.9 ± 48.0	15.2	10.0
Isochlorogenic acid C	1548.2 ± 17.1	11.7	11.1
Neochlorogenic acid	1411.1 ± 22.3	33.1	26.8
Cryptochlorogenic acid	1177.4 ± 10.0	29.1	23.1
Protocatechuic acid	311.5 ± 17.3	17.9	25.3
Caffeic acid	101.7 ± 6.6	22.0	10.8

^1^IC50: the concentration of decreasing the initial DPPH concentration by 50%. ^2^EC50: half effective concentration at which the absorbance was equal to 0.5.

## Data Availability

The Excel data used to support the findings of this study have been deposited in the figshare repository (DOI 10.6084/m9.figshare.11762892).

## Supplementary Materials

Supplementary materials Supplementary material contains some additional data for this paper. Table S1: precursor/product ion pairs and optimized multiple-reaction monitoring (MRM) parameters of phenolic acids. Table S2: relative antioxidant activity and contribution ratio of individual phenolic acid to total antioxidant activity of Wanglaoji herbal tea (WHT) for DPPH scavenging activity. Table S3: relative antioxidant activity and contribution ratio of individual phenolic acid to the antioxidant activity of Wanglaoji herbal tea (WHT) for FRAP activity. Table S4: statistics of reads and mapping rate of RNA-seq data obtained by BGI 500 sequencing. Table S5: expressed change of 271 differentially expressed genes after treatment with Wanglaoji herbal tea compared with the H_2_O_2_-treated group. Table S6: molecular and cellular functions of common differentially expressed genes. Table S7: canonical pathways by which common differentially expressed genes are involved in. Table S8: differentially expressed genes (DEGs) which were regulated by Wanglaoji herbal tea in the opposite direction by regulators from the mechanism network of H_2_O_2_. Table S9: differentially expressed genes (DEGs) which were regulated by Wanglaoji herbal tea in the same direction by regulators from the mechanism network of genistein. Fig. S1: total ion chromatogram of Wanglaoji herbal tea (WHT) and extracted ion chromatograms of six phenolic acids in WHT obtained by multiple-reaction monitoring (MRM) in negative-ion model electrospray ionization mass spectra. Fig. S2: antioxidant activities of six phenolic acids determined by DPPH (A) and FRAP (B) assays at different concentrations. Fig. S3: the viability of cells after treatment with various concentrations of H_2_O_2_. Fig. S4: the viability of cells after treatment with various concentrations of Wanglaoji herbal tea. Fig. S5: mechanism network regulated by H_2_O_2_ from the prediction of Ingenuity Pathway Analysis software. Fig. S6: mechanism network regulated by genistein from the prediction of Ingenuity Pathway Analysis software.
