# Supplementary material for: Antioxidant Effects and Cytoprotective Potentials of Herbal Tea against H2O2-Induced Oxidative Damage by Activating Heme Oxygenase1 Pathway
Source: Biomed Res Int. 2020 Jul 5;2020:7187946. doi: 10.1155/2020/7187946 (PMC7361890; doi:10.1155/2020/7187946)
Supplement: Supplementary materials — Supplementary material contains some additional data for this paper. Table S1: precursor/product ion pairs and optimized multiple-reaction monitoring (MRM) parameters of phenolic acids. Table S2: relative antioxidant activity and contribution ratio of individual phenolic acid to total antioxidant activity of Wanglaoji herbal tea (WHT) for DPPH scavenging activity. Table S3: relative antioxidant activity and contribution ratio of individual phenolic acid to the antioxidant activity of Wanglaoji herbal tea (WHT) for FRAP activity. Table S4: statistics of reads and mapping rate of RNA-seq data obtained by BGI 500 sequencing. Table S5: expressed change of 271 differentially expressed genes after treatment with Wanglaoji herbal tea compared with the H2O2-treated group. Table S6: molecular and cellular functions of common differentially expressed genes. Table S7: canonical pathways by which common differentially expressed genes are involved in. Table S8: differentially expressed genes (DEGs) which were regulated by Wanglaoji herbal tea in the opposite direction by regulators from the mechanism network of H2O2. Table S9: differentially expressed genes (DEGs) which were regulated by Wanglaoji herbal tea in the same direction by regulators from the mechanism network of genistein. Fig. S1: total ion chromatogram of Wanglaoji herbal tea (WHT) and extracted ion chromatograms of six phenolic acids in WHT obtained by multiple-reaction monitoring (MRM) in negative-ion model electrospray ionization mass spectra. Fig. S2: antioxidant activities of six phenolic acids determined by DPPH (A) and FRAP (B) assays at different concentrations. Fig. S3: the viability of cells after treatment with various concentrations of H2O2. Fig. S4: the viability of cells after treatment with various concentrations of Wanglaoji herbal tea. Fig. S5: mechanism network regulated by H2O2 from the prediction of Ingenuity Pathway Analysis software. Fig. S6: mechanism network regulated by genistein from the pr [file 7187946.f1.docx]

**Supplementary Materials**

Supplementary material contains some additional data for this paper. Table S1: Precursor/product ion pairs and optimized multiple-reaction monitoring (MRM) parameters of phenolic acids. Table S2: Relative antioxidant activity and contribution ratio of individual phenolic acid to total antioxidant activity of Wanglaoji herbal tea (WHT) for DPPH scavenging activity. Table S3: Relative antioxidant activity and contribution ratio of individual phenolic acid to the antioxidant activity of Wanglaoji herbal tea (WHT) for FRAP activity. Table S4: Statistics of reads and mapping rate of RNA-seq data obtained by BGI 500 sequencing. Table S5: Express change of 271 differentially expressed genes after treated with Wanglaoji herbal tea compared with H_2_O_2_-treated group. Table S6: Molecular and cellular functions of common differentially expressed genes. Table S7: Canonical pathways which common differentially expressed genes involved in. Table S8: Differentially expressed genes (DEGs) which were regulated by Wanglaoji herbal tea in the opposite direction by regulators from mechanism network of H_2_O_2_. Table S9: Differentially expressed genes (DEGs) which were regulated by Wanglaoji herbal tea in the same direction by regulators from mechanism network of genistein. Fig.S1: Total ion chromatogram of Wanglaoji herbal tea (WHT) and extracted ion chromatograms of six phenolic acids in WHT obtained by multiple-reaction monitoring (MRM) in negative–ion model electrospray ionization mass spectra. Fig. S2: Antioxidant activities of six phenolic acids determined by DPPH (A) and FRAP (B) assays at different concentrations. Fig. S3: The viability of cells after treated with various concentration of H_2_O_2_. Fig. S4: The viability of cells after treated with various concentration of Wanglaoji herbal tea. Fig. S5: Mechanism network regulated by H_2_O_2_ from the prediction of Ingenuity Pathway Analysis software. Fig. S6: Mechanism network regulated by genistein from the prediction of Ingenuity Pathway Analysis software.

Supplementary Table S1**.** Precursor/product ion pairs and optimized multiple-reaction monitoring (MRM) parameters of phenolic acids

| Compounds | Precursor  ion (m/z) | Production  ion (m/z) | Fragmentor  (V) | Collision  energy (V) |
| --- | --- | --- | --- | --- |
| Rosmarinic acid | 359.1 | 197/160.8^a^ | 120 | 14/22 |
| Caffeic acid | 179.1 | 134^a^/88.8 | 100 | 28/28 |
| Protocatechuic acid | 153.0 | 109.1^a^/91 | 90 | 32/30 |
| Isochlorogenic acid C | 515.2 | 352.9^a^/172.8 | 130 | 16/40 |
| Cryptochlorogenic acid | 353.1 | 173^a^/134.9 | 110 | 10/36 |
| Neochlorogenic acid | 353.1 | 191^a^/134.9 | 100 | 16/34 |

^a^ Production ion for quantitation

Supplementary Table S2**.** Relative antioxidant activity and contribution ratio of individual phenolic acid to total antioxidant activity of Wanglaoji herbal tea (WHT) for DPPH scavenging activity

| Sample | IC50  (µg/mL) | Relative  antioxidant activity^1^ | Content  (µg/g) | Contribution  Ratio^2^ |
| --- | --- | --- | --- | --- |
| Rosmarinic acid | 5.5 | 14.0 | 3426.9 | 4.8 |
| Caffeic acid | 4.0 | 19.3 | 101.7 | 0.2 |
| Protocatachuic acid | 2.8 | 27.7 | 311.5 | 0.9 |
| Isochlorogenic acid C | 6.0 | 12.7 | 1548.2 | 2.0 |
| Cryptochlorogenic acid | 10.3 | 7.4 | 1177.4 | 0.9 |
| Neochlorogenic acid | 11.7 | 6.5 | 1411.1 | 0.9 |
| WHT | 76.6 | 1.0 | 1000000 | 100.0 |

IC50, the concentration of decreasing the initial DPPH concentration by 50%

^1^ the ratio of the IC50 value of WHT to IC50 value of individual phenolic acid

^2^ Relative antioxidant activity* content/1000000*100%

Supplementary Table S3**.** Relative antioxidant activity and contribution ratio of individual phenolic acid to the antioxidant activity of Wanglaoji herbal tea (WHT) for FRAP activity

| Sample | EC50  (µg/mL) | Relative  antioxidant activity^1^ | Content  (µg/g) | Contribution  Ratio^2^ |
| --- | --- | --- | --- | --- |
| Rosmarinic acid | 1.6 | 24.4 | 3426.9 | 8.3 |
| Caffeic acid | 1.0 | 39.0 | 101.7 | 0.4 |
| Protocatachuic acid | 1.9 | 19.6 | 311.5 | 0.6 |
| Isochlorogenic c | 2.8 | 13.5 | 1548.2 | 2.1 |
| Cryptochlorogenic acid | 4.1 | 9.2 | 1177.4 | 1.1 |
| Neochlorogenic acid | 4.8 | 7.9 | 1411.1 | 1.1 |
| WHT | 38.1 | 1.0 | 1000000 | 100.0 |

EC50, half effective concentration at which the absorbance is equal to 0.5

^1^ the ratio of the EC50 value of WHT to EC50 value of individual phenolic acid

^2^ Relative antioxidant activity* content/1000000*100%

Supplementary Table S4. Statistics of reads and mapping rate of RNA-seq data obtained by BGI 500 sequencing

| Group | Total raw reads (M) | Clean reads ratio(%) | Total mapping(%) |
| --- | --- | --- | --- |
| Control_1 | 21.7 | 98.4 | 95.4 |
| Control_2 | 21.7 | 98.4 | 95.4 |
| Control_3 | 21.7 | 98.3 | 95.5 |
| H_2_O_2__1 | 21.7 | 98.4 | 95.4 |
| H_2_O_2__2 | 21.7 | 98.4 | 95.2 |
| H_2_O_2__3 | 21.7 | 98.3 | 95.2 |
| WHT+ H_2_O_2__1 | 21.9 | 97.0 | 95.4 |
| WHT+ H_2_O_2__2 | 21.9 | 97.2 | 95.3 |
| WHT+ H_2_O_2__3 | 21.9 | 96.7 | 95.2 |
| Average | 21.8 | 97.9 | 95.3 |

WHT, Wanglaoji herb tea;

Supplementary Table S5. Express change of 271 differentially expressed genes after treated with Wanglaoji herbal tea compared with H_2_O_2_-treated group

| No | Genes | Level | No | Genes | Level | No | Genes | Level |
| --- | --- | --- | --- | --- | --- | --- | --- | --- |
| 1 | TRIB3 | + | 41 | GDNF | + | 81 | PSMB8 | + |
| 2 | PLAAT4 | + | 42 | AKR1C1 | + | 82 | PIR | + |
| 3 | MTRNR2L3 | + | 43 | TMEM206 | + | 83 | FBXO25 | + |
| 4 | GDF15 | + | 44 | ARHGAP45 | + | 84 | MOCOS | + |
| 5 | HIST1H2BC | + | 45 | MSC | + | 85 | PARD6G | + |
| 6 | DDIT4 | + | 46 | C15orf40 | + | 86 | TMEM38B | + |
| 7 | HEXIM2 | + | 47 | BLOC1S4 | + | 87 | PIDD1 | + |
| 8 | ATF3 | + | 48 | MTRNR2L6 | + | 88 | HIST1H2AC | + |
| 9 | DDIT3 | + | 49 | NCOA7 | + | 89 | KLHDC2 | + |
| 10 | LAGE3 | + | 50 | BLVRB | + | 90 | RAB9A | + |
| 11 | UNC5B | + | 51 | ECH1 | + | 91 | PSMB9 | + |
| 12 | INHBE | + | 52 | B3GNTL1 | + | 92 | HEXA | + |
| 13 | CXCL3 | + | 53 | ABHD8 | + | 93 | BBS1 | + |
| 14 | TRAPPC6A | + | 54 | CREG1 | + | 94 | EIF4EBP1 | + |
| 15 | ABHD16B | + | 55 | GNPDA1 | + | 95 | NTPCR | + |
| 16 | MTHFD2 | + | 56 | LRIG1 | + | 96 | QPCT | + |
| 17 | MYL5 | + | 57 | CADM1 | + | 97 | SLC36A4 | + |
| 18 | USP31 | + | 58 | HDAC11 | + | 98 | ABCB10 | + |
| 19 | KLHL13 | + | 59 | DUT | + | 99 | NAPRT | + |
| 20 | HMOX1 | + | 60 | TMEM268 | + | 100 | TMEM160 | + |
| 21 | TRIM16L | + | 61 | STC2 | + | 101 | NMI | + |
| 22 | IL6 | + | 62 | DECR2 | + | 102 | MAGOHB | + |
| 23 | CLDN23 | + | 63 | LOC102723872 | + | 103 | RPS27 | + |
| 24 | LOC107985476 | + | 64 | TRIAP1 | + | 104 | FAM212B | + |
| 25 | GPNMB | + | 65 | GLI4 | + | 105 | TSC22D3 | + |
| 26 | ZNF853 | + | 66 | TMEM65 | + | 106 | XPOT | + |
| 27 | KIAA1644 | + | 67 | BAMBI | + | 107 | THNSL2 | + |
| 28 | EPM2A | + | 68 | TMEM128 | + | 108 | STOM | + |
| 29 | KCNE4 | + | 69 | SDSL | + | 109 | RAB24 | + |
| 30 | HEXDC | + | 70 | CCDC28A | + | 110 | KYNU | + |
| 31 | NIBAN1 | + | 71 | C6orf48 | + | 111 | MORN4 | + |
| 32 | MTRNR2L9 | + | 72 | ZNF32 | + | 112 | RRAGB | + |
| 33 | PLAAT3 | + | 73 | WDYHV1 | + | 113 | NARS2 | + |
| 34 | CCDC61 | + | 74 | BBC3 | + | 114 | DUSP22 | + |
| 35 | RGS17 | + | 75 | FMNL1 | + | 115 | H1-2 | + |
| 36 | PHYH | + | 76 | ISOC1 | + | 116 | MPPE1 | + |
| 37 | GLRB | + | 77 | FAM110A | + | 117 | KRT10 | + |
| 38 | CHEK2 | + | 78 | SESN1 | + | 118 | MTURN | + |
| 39 | CEBPG | + | 79 | DNMT3A | + | 119 | THYN1 | + |
| 40 | TCN2 | + | 80 | TATDN3 | + | 120 | STARD5 | + |

Supplementary Table S5. Express change of 271 differentially expressed genes after treated with Wanglaoji herbal tea compared with H_2_O_2_-treated group (continued)

| No | Genes | Level | No | Genes | Level | No | Genes | Level |
| --- | --- | --- | --- | --- | --- | --- | --- | --- |
| 121 | PKDCC | + | 161 | FAM198B | + | 201 | NECTIN1 | - |
| 122 | MOXD1 | + | 162 | ASAH1 | + | 202 | THEMIS2 | - |
| 123 | RMND5A | + | 163 | BDH2 | + | 203 | LOC101060022 | - |
| 124 | MAD2L2 | + | 164 | ZNF197 | - | 204 | RNF152 | - |
| 125 | ALPK1 | + | 165 | ECHDC2 | - | 205 | PLEKHG2 | - |
| 126 | TIMM10 | + | 166 | INO80B | - | 206 | DRAP1 | - |
| 127 | CMBL | + | 167 | RPS6KA6 | - | 207 | XYLT1 | - |
| 128 | C2orf69 | + | 168 | MTF1 | - | 208 | GABBR1 | - |
| 129 | NINJ1 | + | 169 | CAVIN1 | - | 209 | TM4SF1 | - |
| 130 | PIM3 | + | 170 | WDR91 | - | 210 | CENPJ | - |
| 131 | FBXO22 | + | 171 | PLD2 | - | 211 | C6orf132 | - |
| 132 | MOSPD1 | + | 172 | YAE1D1 | - | 212 | ETV1 | - |
| 133 | C19orf48 | + | 173 | BRI3BP | - | 213 | TRMT44 | - |
| 134 | HTATIP2 | + | 174 | TPM2 | - | 214 | DDAH1 | - |
| 135 | ORAI3 | + | 175 | LCOR | - | 215 | DPF1 | - |
| 136 | DNPEP | + | 176 | AUTS2 | - | 216 | RECQL4 | - |
| 137 | FEZ1 | + | 177 | WWC1 | - | 217 | APOOL | - |
| 138 | CEP57 | + | 178 | MNT | - | 218 | MGAT5 | - |
| 139 | RSL24D1 | + | 179 | C15orf52 | - | 219 | COL7A1 | - |
| 140 | PCBP3 | + | 180 | LOC107984841 | - | 220 | PCDHGC3 | - |
| 141 | HERPUD1 | + | 181 | ARHGAP18 | - | 221 | CORO6 | - |
| 142 | ALKBH3 | + | 182 | ZWILCH | - | 222 | CABIN1 | - |
| 143 | WARS1 | + | 183 | TNS2 | - | 223 | COL27A1 | - |
| 144 | LSM5 | + | 184 | MAPK13 | - | 224 | NEGR1 | - |
| 145 | PPP1R3C | + | 185 | PARP15 | - | 225 | CDH24 | - |
| 146 | ATRAID | + | 186 | LDLR | - | 226 | SMTN | - |
| 147 | RBM43 | + | 187 | GJC1 | - | 227 | SHROOM3 | - |
| 148 | TKFC | + | 188 | HS3ST3A1 | - | 228 | INHBA | - |
| 149 | COX7A2L | + | 189 | TMEM106A | - | 229 | POLR3G | - |
| 150 | FAM58A | + | 190 | YJEFN3 | - | 230 | SYTL5 | - |
| 151 | TDRD3 | + | 191 | ARHGDIA | - | 231 | MSN | - |
| 152 | CYB5A | + | 192 | HDAC5 | - | 232 | UACA | - |
| 153 | BTBD6 | + | 193 | PDLIM5 | - | 233 | DRP2 | - |
| 154 | NFE2L2 | + | 194 | ZC3H18 | - | 234 | RPL39L | - |
| 155 | SRXN1 | + | 195 | LIMS2 | - | 235 | NEK10 | - |
| 156 | TIPARP | + | 196 | SYNM | - | 236 | ZNF350 | - |
| 157 | EIF4A2 | + | 197 | ZNF415 | - | 237 | LMO7 | - |
| 158 | RASSF1 | + | 198 | MDC1 | - | 238 | TFEB | - |
| 159 | HINT3 | + | 199 | KCND1 | - | 239 | AURKA | - |
| 160 | NAMPT | + | 200 | FOXM1 | - | 240 | NRBP2 | - |

Supplementary Table S5. Express change of 271 differentially expressed genes after treated with Wanglaoji herbal tea compared with H_2_O_2_-treated group (continued)

| No | Genes | Level | No | Genes | Level | No | Genes |
| --- | --- | --- | --- | --- | --- | --- | --- |
| 241 | LOC102723728 | - | 252 | PTGIR | - | 263 | TRIP13 |
| 242 | SHANK1 | - | 253 | CARD6 | - | 264 | MMP3 |
| 243 | PNPLA3 | - | 254 | GNB3 | - | 265 | MMP1 |
| 244 | KCNMA1 | - | 255 | MARCH4 | - | 266 | APCDD1L |
| 245 | LOC107985355 | - | 256 | NUP35 | - | 267 | COL13A1 |
| 246 | IGF2 | - | 257 | LETM2 | - | 268 | LOC107986800 |
| 247 | PLGLB2 | - | 258 | IL13RA2 | - | 269 | KIAA1549L |
| 248 | DSP | - | 259 | ADAP2 | - | 270 | STC1 |
| 249 | TOP2A | - | 260 | LIPT1 | - | 271 | RGS4 |
| 250 | DLL4 | - | 261 | TGM2 | - |  |  |
| 251 | RAB3B | - | 262 | MMP16 | - |  |  |

+, upregulation;-, downregulation

Supplementary Table S6. Molecular and cellular functions of common differentially expressed genes

| Molecular and cellular functions | Gene |
| --- | --- |
| Cell cycle | AURKA, CHEK2, FOXM1, RASSF1, TOP2A, CEBPG, DDIT3, IL6,  TRIB3, GDF15, NFE2L2, TSC22D3, INHBA, MAD2L2, CENPJ, IGF2, ORAI3, SYNM, TRIP13, CREG1, HMOX1, ATF3, ARHGAP18, PIM3, EIF4EBP1 |
| Cellular compromise | DDIT3, DLL4, HMOX1, IGF2, IL6, NFE2L2, PIM3, ATF3, BBC3, HERPUD1, NIBAN1, STC2, TRIB3, CXCL3, EPM2A, TGM2, NAMPT, ARHGDIA, CADM1, GDNF, KCNMA1, LDLR |
| Cell death and survival | ALKBH3, ARHGAP18, ARHGDIA, ASAH1, ATF3, AURKA, BAMBI, BBC3, CABIN1, CADM1, CENPJ, CHEK2, COX7A2L, CREG1, CXCL3, CYB5A, DDIT3, DDIT4, DLL4, DNMT3A, DPF1, DSP, DUSP22, DUT, EIF4EBP1, EPM2A, FBXO25, FOXM1, GABBR1, GDF15, GDNF, GJC1, GPNMB, H1-2, HDAC11, HDAC5, HERPUD1, HMOX1, HTATIP2, MNT, IGF2, IL6, INHBA, KCNMA1, LDLR, LIMS2, LRIG1, MAD2L2, MAPK13, MDC1, MGAT5, MMP1, MMP3, NMI, MSN, MTF1, NAMPT, NCOA7, NFE2L2, NINJ1, NRBP2, PCDHGC3, PIDD1, PIM3, PLAAT3, PLAAT4, PLD2, PLEKHG2. PSMB9, RASSF1, RBM43, RECQL4, RGS4, RNF152, RPS6KA6, SRXN1, STC1, SYNM, TFEB, TGM2, TNS2, TOP2A, TRIAP1, TRIB3, TSC22D3, UACA, UNC5B, WWC1 |
| Cellular movement | ALKBH3, ARHGAP18, ARHGDIA, ATF3, AURKA, AUTS2, BBC3, BBS1, CADM1, COL7A1, CXCL3, DDIT4, DLL4, DNMT3A, IGF2, DSP, DUSP22, EIF4EBP1, ETV1, FOXM1, GDF15, GDNF, GLRB, GPNMB, HMOX1, HTATIP2, IL13RA2, IL6, INHBA, KCNMA1, KRT10, LDLR, LIMS2, LMO7, LRIG1, MAD2L2, MAPK13, MGAT5, MMP1, MMP16, MMP3, MSN, NAMPT, NFE2L2, NINJ1, PLAAT3, PLD2, PLEKHG2, PSMB9, PTGIR, QPCT, RASSF1, RGS4, RPS6KA6, STC1, SYNM, TGM2, TM4SF1,TNS2, TSC22D3, UNC5B, WARS1, WWC1, ZNF350 |
| Gene expression | ATF3, ATRAID, AUTS2, BAMBI, CAVIN1, CEBPG, CENPJ, CHEK2, CREG1, CXCL3, DDIT3, DLL4, DNMT3A, DRAP1, DUSP22, EIF4A2, EIF4EBP1, EPM2A, ETV1, FOXM1, GDNF, GNB3, H1-2, HDAC5, MSC, HEXIM2, HTATIP2, IGF2, IL6, INHBA, LCOR, LDLR, LMO7, MAD2L2, MNT, MOSPD1, MSN, MTF1, NAMPT, NCOA7, NFE2L2, NMI, PARP15, PCBP3, PIR, PLAAT4, PLEKHG2, POLR3G, PTGIR, STC2, TFEB, TGM2, TIPARP, TOP2A, TRIAP1, TRIB3, TRIP13, TSC22D3, WARS1, WWC1, ZNF350 |

Supplementary Table S7. Canonical pathways which common differentially expressed genes involved in

| Canonical pathways | Genes |
| --- | --- |
| Phospholipases | HMOX1, PLAAT3, PLAAT4, PLD2, PNPLA3 |
| IL-10 Signaling | BLVRB, HMOX1, IL6, MAPK13 |
| mTOR signaling | DDIT4, EIF4A2, EIF4EBP1, HMOX1, PLD2, RPS27, RPS6KA6 |
| EIF2 signaling | ATF3, DDIT3, EIF4A2, RPL39L, RPS27, TRIB3, WARS1 |
| Aryl Hydrocarbon receptor signaling | CHEK2, IL6, NCOA7, NFE2L2, TGM2 |
| Leukoyte extravasation signaling | CLDN23, MAPK13, MMP1, MMP3, MMP16, MSN |
| Phospholipases c signaling | GNB3, HDAC11, HMOX1, MYL5, PLD2, TGM2 |

Supplementary Table S8. Differentially expressed genes (DEGs) which were regulated by Wanglaoji herbal tea in the opposite direction by regulators from mechanism network of H_2_O_2_

| DEGs | Gene  change | Regulators from mechanism network | | | | | | | | |
| --- | --- | --- | --- | --- | --- | --- | --- | --- | --- | --- |
|  |  | H_2_O_2_ | NFkB  (Complex)^a^ | RELA^a^ | HIF1A^a^ | DDIT3^a^ | TP53^a^ | ATF4^a^ | FOXO3^a^ | E2F1^b^ |
| TRIB3 | **+** |  |  |  |  |  | **-** |  |  |  |
| CXCL3 | **+** |  |  |  |  |  | **-** |  |  |  |
| UNC5B | **+** |  |  |  |  |  | **-** |  |  |  |
| DDIT3 | **+** |  |  | **-** |  |  |  |  |  |  |
| CHEK2 | **+** |  |  |  |  |  | **-** |  |  |  |
| IL6 | **+** |  |  |  |  |  | **-** |  | **-** |  |
| MTHFD2 | **+** |  |  |  |  | **-** |  |  | **-** |  |
| HMOX1 | **+** |  | **-** |  |  |  |  |  |  |  |
| DUT | **+** |  |  |  |  |  | **-** |  |  |  |
| DNMT3A | **+** |  |  |  |  |  | **-** |  |  |  |
| CYB5A | **+** |  |  |  | **-** |  |  |  |  |  |
| BBC3 | **+** |  |  |  |  |  |  |  |  | **+** |
| TSC22D3 | **+** |  |  |  |  | **-** |  |  |  |  |
| BAMBI | **+** |  | **-** |  |  |  |  |  |  |  |
| TIMM10 | **+** |  |  |  |  |  | **-** |  |  |  |
| RECQL4 | **-** |  |  |  |  |  | **+** |  |  |  |
| FOXM1 | **-** |  |  |  |  |  | **+** |  | **+** |  |
| IGF2 | **-** |  |  |  |  |  |  |  |  | **-** |
| TGM2 | **-** |  |  |  |  | **+** |  |  |  |  |
| MMP16 | **-** |  |  |  |  |  |  |  |  | **-** |
| AURKA | **-** | **+** |  |  |  |  | **+** |  |  | **-** |
| MMP1 | **-** |  |  |  |  |  | **+** |  |  |  |
| TOP2A | **-** |  |  |  |  |  | **+** |  |  |  |
| MMP3 | **-** |  |  |  |  |  | **+** |  |  |  |
| RGS4 | **-** |  |  |  |  | **+** |  |  |  |  |

+, upregulation or activated; -, downregulation or inhibited.

a, activated by H_2_O_2_; b, inhibited by H_2_O_2_

Supplementary Table S9. Differentially expressed genes (DEGs) which were regulated by Wanglaoji herbal tea in the same direction by regulators from mechanism network of genistein

| DEGs | Gene  change | Regulators from mechanism network | | | | |
| --- | --- | --- | --- | --- | --- | --- |
|  |  | Genistein | Resveratrol | RELA^a^ | DDIT3^a^ | TP53^b^ |
| PLAAT4 | **+** |  |  |  |  | **+** |
| GDF15 | **+** | **+** | **+** |  |  | **+** |
| DDIT3 | **+** |  |  | **-** |  | **+** |
| ATF3 | **+** |  | **+** |  |  | **+** |
| DDIT4 | **+** |  |  |  |  | **+** |
| HMOX1 | **+** | **+** | **+** |  |  | **+** |
| HEXD | **+** | **+** |  |  |  |  |
| PIDD1 | **+** |  |  |  |  | **+** |
| ECH1 | **+** |  |  |  |  | **+** |
| NINJ1 | **+** |  |  |  |  | **+** |
| HEXA | **+** | **+** |  |  |  |  |
| STOM | **+** |  |  |  |  | **+** |
| CMBL | **+** |  |  |  |  | **+** |
| TRIAP1 | **+** |  |  |  |  | **+** |
| CYB5A | **+** |  |  |  |  | **+** |
| BBC3 | **+** |  |  |  |  | **+** |
| TSC22D3 | **+** |  |  |  | **-** | **+** |
| PARD6G | **+** |  |  |  |  | **+** |
| SESN1 | **+** |  |  |  |  | **+** |
| NAMPT | **+** |  |  |  |  | **+** |
| FBXO22 | **+** |  |  |  |  | **+** |
| NFE2L2 | **+** | **+** |  |  |  |  |
| ASAH1 | **+** | **+** |  |  |  |  |
| HDAC5 | **-** |  |  |  |  | **-** |
| PDLIM5 | **-** |  |  |  |  | **-** |
| COL7A1 | **-** |  |  |  |  | **-** |
| LDLR | **-** | **-** |  |  |  | **-** |
| SHROOM3 | **-** |  |  |  |  | **-** |
| IGF2 | **-** |  | **-** |  |  | **-** |
| MDC1 | **-** | **-** |  |  |  |  |
| TFEB | **-** | **-** |  |  |  |  |
| INHBA | **-** |  |  |  |  | **-** |
| KCNMA1 | **-** |  |  |  |  | **-** |
| TGM2 | **-** |  |  |  | **+** | **-** |
| COL13A1 | **-** |  |  |  |  | **-** |

+, upregulation or activated; -, downregulation or inhibited.

a, activated by genistein; b, inhibited by genistein

Supplementary Fig.S1


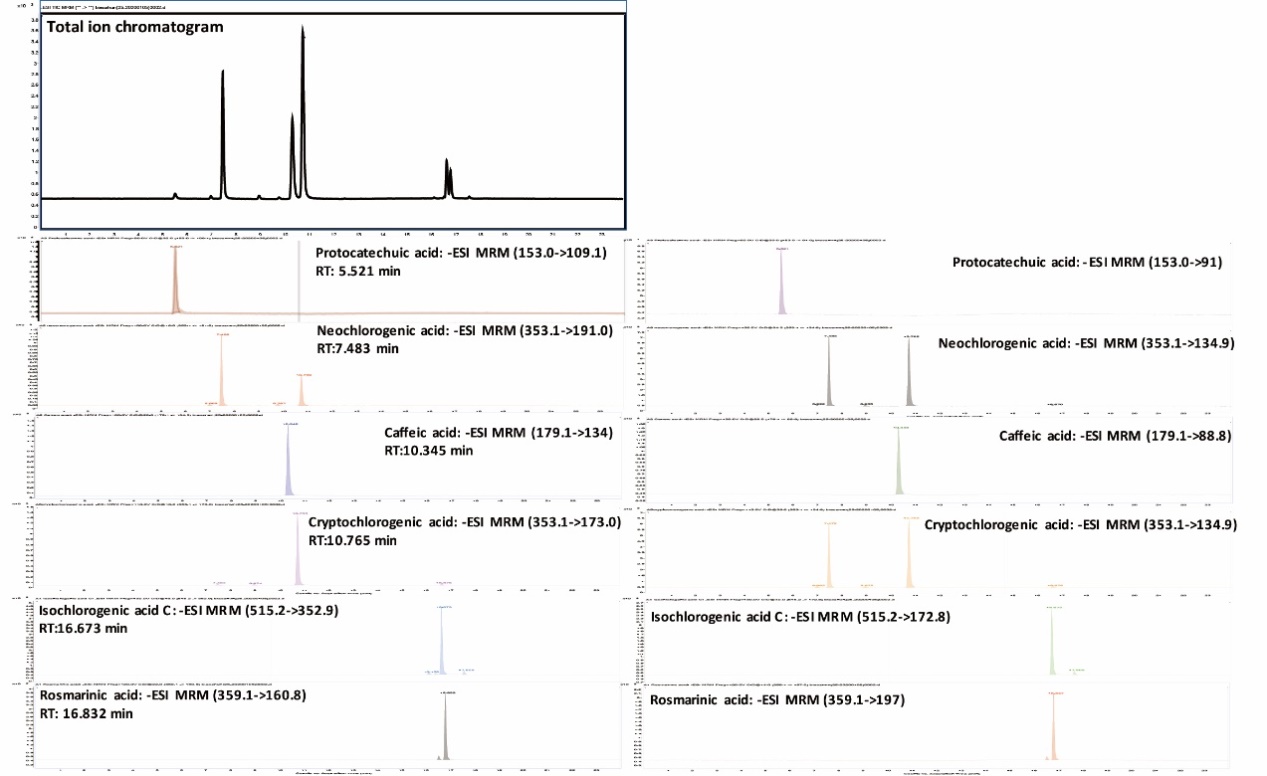


Fig S1. Total ion chromatogram of Wanglaoji herbal tea (WHT) and extracted ion chromatograms of six phenolic acids in WHT obtained by multiple-reaction monitoring (MRM) in negative–ion model electrospray ionization mass spectra

Supplementary Fig.S2


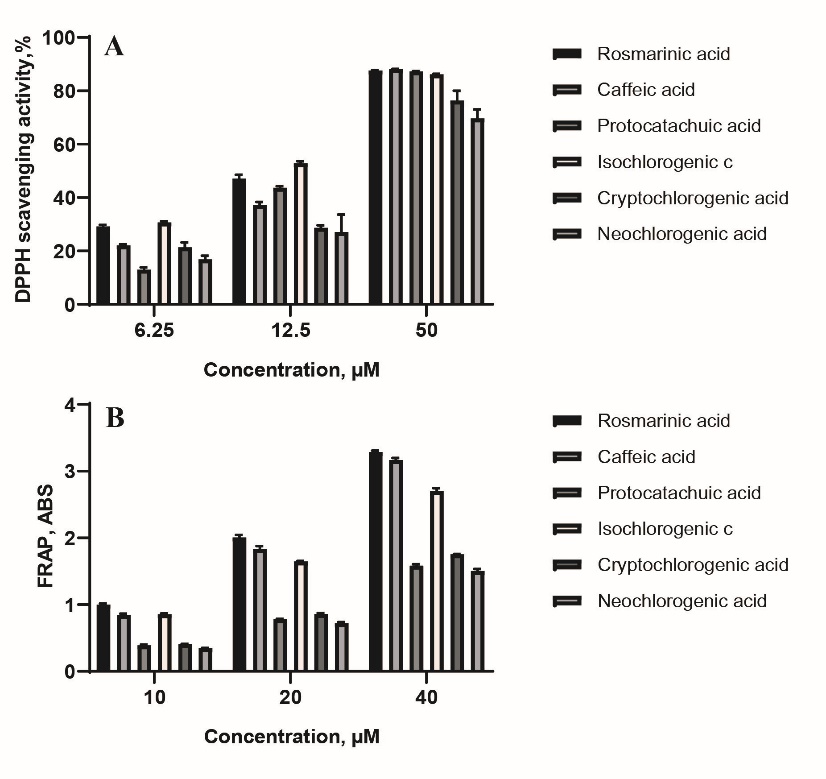


Fig S2. Antioxidant activities of six phenolic acids determined by DPPH (A) and FRAP (B) assays at different concentrations.

Supplementary Fig.S3


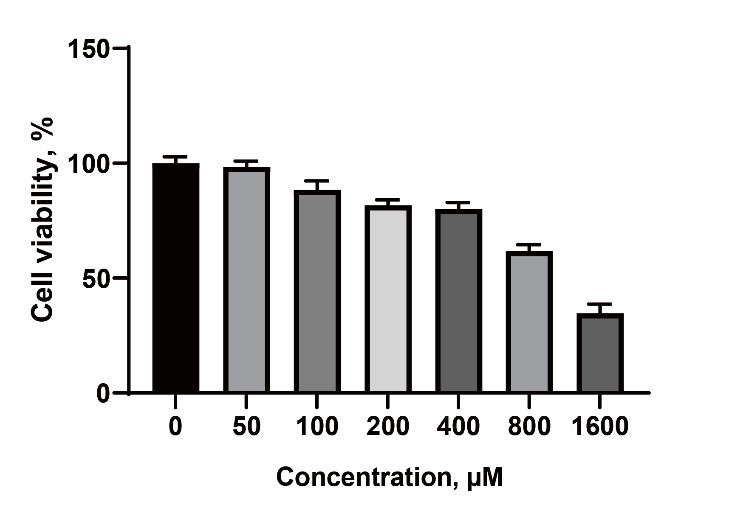


Fig S3. The viability of cells after treated with various concentration of H_2_O_2_

Supplementary Fig.S4


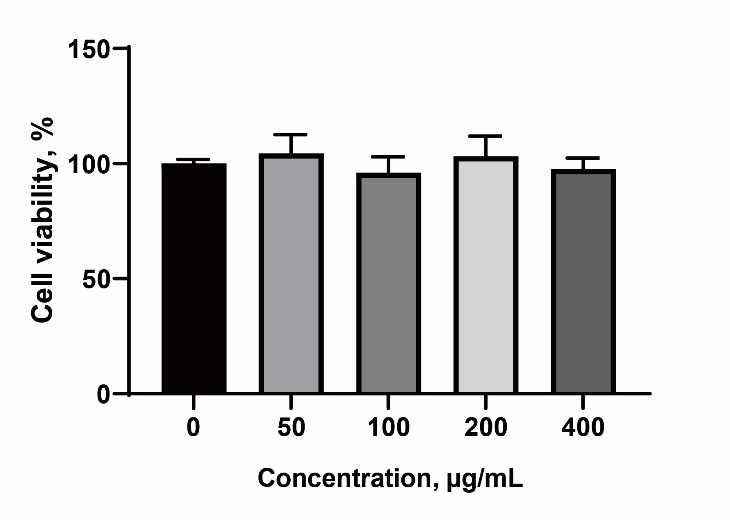


Fig S4. The viability of cells after treated with various concentration of Wanglaoji herbal tea

Supplementary Fig.S5


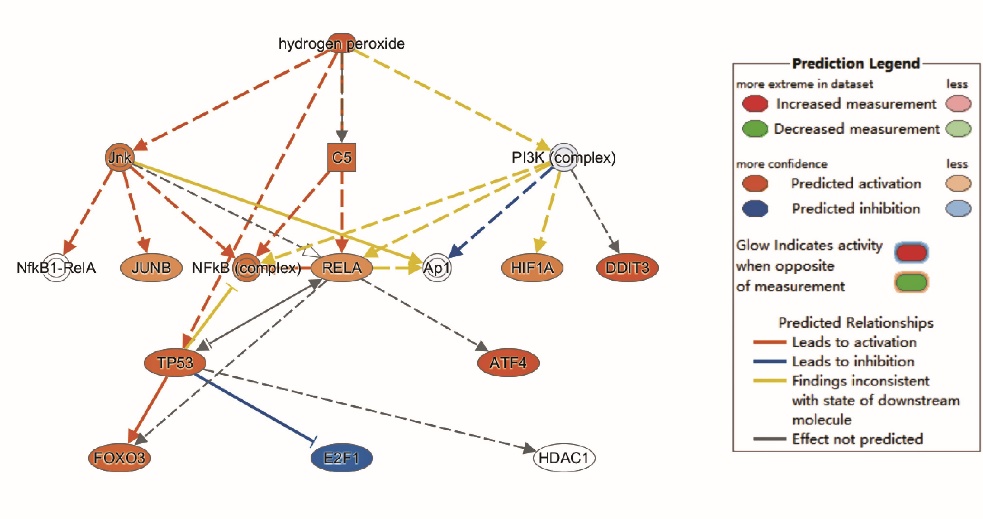


Fig S5**.** Mechanism network regulated by H_2_O_2_ from the prediction of Ingenuity Pathway Analysis software

Supplementary Fig.S6


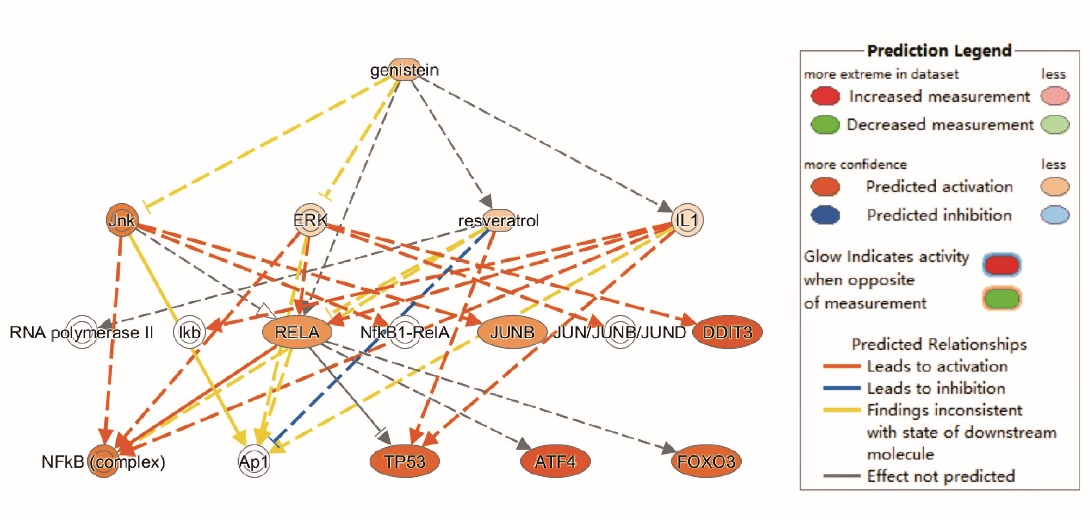


Fig S6**.** Mechanism network regulated by genistein from the prediction of Ingenuity Pathway Analysis software
